# Comparison of the diagnostic value between triglyceride-glucose index and triglyceride to high-density lipoprotein cholesterol ratio in metabolic-associated fatty liver disease patients: a retrospective cross-sectional study

**DOI:** 10.1186/s12944-022-01661-7

**Published:** 2022-06-25

**Authors:** Zhi Liu, He He, Yuzhao Dai, Lidan Yang, Shenling Liao, Zhenmei An, Shuangqing Li

**Affiliations:** 1 Department of Laboratory Medicine, West China Hospital, Sichuan University, Chengdu, 610041 Sichuan China; 2 Department of Endocrinology and Metabolism, West China Hospital, Sichuan University, Chengdu, 610041 Sichuan China; 3 Department of General Practice, West China Hospital, Sichuan University, Chengdu, 610041 Sichuan China

## Abstract

**Background:**

The triglyceride and glucose index (TyG) and triglyceride to high-density lipoprotein cholesterol ratio (TG/HDL-C) are substitute markers of insulin resistance (IR). In a retrospective cross-sectional study, the authors aimed to compare the efficacy of the two indicators in diagnosing metabolic-associated fatty liver disease (MAFLD) to construct a novel disease diagnosis model.

**Methods:**

Overall, 229 patients (97 MAFLD and 132 Non-MAFLD at West China Hospital of Sichuan University were included. MAFLD was diagnosed using ultrasonography. Biochemical indexes were collected and analyzed by logistic regression to screen out indicators that were expressed differently in MAFLD patients and healthy controls, which were incorporated into a diagnostic model.

**Results:**

After adjusting for age, sex, and body mass index (BMI), serum alanine transaminase (ALT), aspartate transaminase (AST), AST/ALT (A/A), fasting plasma glucose (FPG), cystatin C (Cys-C), uric acid (URIC), triglycerides (TG), high-density lipoprotein cholesterol (HDL-C), alkaline phosphatase (ALP), gamma-glutamyl transferase (GGT), non-HDL-C, LDL-C/HDL-C, non-HDL-C/HDL-C, TG/HDL-C, TC/HDL-C, TyG, and TyG-BMI were risk factors for MAFLD. The odds ratio of TG/HDL-C and TyG were 5.629 (95%CI: 3.039–10.424) and 182.474 (95%CI: 33.518–993.407), respectively. In identifying MAFLD, TyG, TyG-BMI, TG, and TG/HDL-C were found to be the most vital indexes based on the random forest method, with the area under the curve (AUC) greater than 0.9. In addition, the combination of BMI, ALT, and TyG had a high diagnostic efficiency for MAFLD.

**Conclusions:**

TyG and TG/HDL-C were potential risk factors for MAFLD, and the former performed better in diagnosing MAFLD. The combination of BMI, ALT, and TyG improved the diagnostic capability for MAFLD.

## Background

Considering the current understanding of its pathogenesis and its rising prevalence, nonalcoholic fatty liver disease (NAFLD) is now regarded as metabolic-associated fatty liver disease (MAFLD) [1]. Different diagnostic criteria did not affect the prevalence of NAFLD or MAFLD in the United States (US) [2]. Affecting more than a quarter of adults worldwide, MAFLD is becoming one of the most common liver diseases [3]. The incidence of MAFLD continues to increase in developed countries such as the US and European countries like Germany, France, Italy, and the United Kingdom, causing a tremendous economic burden [4]. In China, a significant increase in the occurrence of MAFLD is expected, from 243.66 million in 2016 to 314.58 million in 2030 [5]. In China, several challenges associated with MAFLD are as follows: a large number of cases, high genetic susceptibility, high occurrence in young patients, absence of attention and recognition, and the lack of adequate diagnostic methods and treatments [6].

Although the pathogenesis theory of MAFLD has changed from the “two-hit” theory to the “multi-hit” theory, insulin resistance (IR) still plays a significant role in the development of MAFLD [7]. Regarded as the gold standard method to measure IR, hyperinsulinemic-euglycemic clamp (HIEC) is complicated, time-consuming, and expensive. Triglyceride to high-density lipoprotein cholesterol (TG/HDL-C) and triglyceride and glucose index (TyG) have been shown to be useful biomarkers for identifying individuals with IR in a large group of Chinese individuals [8, 9]. Early in 2005, TG/HDL-C was considered an important factor in predicting IR and was shown to increase the risk of cardiovascular diseases in patients [10]. In addition to race, the optimum cut-off value of TG/HDL-C for predicting IR was different between genders (female > 2.5, male > 3.5), and patients with TG/HDL-C above the cut-off value were exposed to higher cardiovascular diseases (CVD) risk [11]. Both TG/HDL-C and metabolic syndrome (MS) can effectively diagnose IR and predict the risk of CVD, while TyG is relatively weak in predicting the occurrence of CVD [12–14]. TG/HDL-C is also correlated with the occurrence of diabetes mellitus (DM) with related vascular diseases and fatty liver disease [15–17]. TyG, calculated using fasting plasma glucose (FPG) and triglycerides (TG), has a close connection with IR, which is related to glucolipid metabolism [18]. In detecting IR, TyG is cheaper and more convenient than the homeostasis model assessment of insulin resistance (HOMA-IR) index, which is commonly used as a substitute for the HIEC [19]. TyG is also associated with cardiometabolic diseases and is a risk prognostic factor for stroke, DM, acute myocardial infarction, and acute coronary syndrome [20–22]. In addition, TyG may be a useful indicator of MAFLD not only in adults but also in children and the elderly [23–25]. Therefore, this study aimed to compare the ability of TG/HDL-C and TyG to distinguish MAFLD from healthy people and establish a better prediction model for MAFLD.

## Methods

Overall, 229 participants were enrolled from West China Hospital between October 2018 and March 2021, including 97 patients with MAFLD (MAFLD group) and 132 individuals who underwent a physical examination (non-MAFLD group). MAFLD was diagnosed by experienced clinicians based on the abdominal ultrasound diagnosis of hepatic steatosis and evidence of any of the following three conditions: overweight/obesity, type 2 diabetes mellitus (T2DM), or metabolic disorders [26]. Since MAFLD emphasizes the influence of metabolic factors and can coexist with other liver diseases, MAFLD patients included in our study did not exclude patients with liver diseases caused by excessive alcohol consumption and viruses [27, 28].

Fasting blood samples from the median cubital vein were used to quantify total bilirubin (TBIL), indirect bilirubin (IBIL), direct bilirubin (DBIL), ALT, AST, total protein (TP), albumin (ALB), globulin (GLB), ALP, FPG, TG, total cholesterol (TC), TG, HDL-C, low-density lipoprotein cholesterol (LDL-C), URIC, urea (UREA), creatinine (CREA), Cys-C, creatine kinase (CK), lactate dehydrogenase (LDH), and hydroxybutyrate dehydrogenase (HBDH) using Roche’s automatic biochemical analyzer and the corresponding kit (Roche, Mannheim, Germany). In this study, TyG was calculated using established formulas: TyG = Ln [TG (mg/dl) × FPG (mg/dl)/2] [19]. The formula used for converting mmol/L to mg/dL is as follows: for FPG, 1 mmol/L = 18 mg/dL; for TG, 1 mmol/L = 88.5 mg/dL. TyG-BMI was equal to TyG index × BMI. Hepatic steatosis index (HSI) = 8 × (ALT/AST ratio) + BMI (+ 2, if female; + 2, if diabetes mellitus).

Statistical analysis and graph drawing was done using SPSS 22.0 (IBM, Corp., N.Y., USA) and R software 4.1.1 (R Foundation for Statistical Computing, Vienna, Austria). Student’s t-test and Mann-Whitney U test were used to compare two groups of normal or non-normal distributed continuous variables through the *tableone* package of the R language, respectively. Through SPSS 22.0, Pearson’s chi-square test was used for categorical variables, and logistic regression analysis was used to identify independent risk factors for MAFLD with the OR value expressed with a 95% confidence interval (CI). After constructing a new predictive MAFLD model by binary logistic regression, the model was graphed by nomogram through the *rms* package. A receiver operating characteristic curve (ROC curve) through the *pROC* package was used for diagnostic value analysis, and the maximum value of the Youden index (sensitivity+specificity-1) was taken as the optimal cut-off value. Cross-validation was used to describe the predictive efficacy of the model by *tidyverse* and *caret* packages. Hosmer-Lemeshow test and calibration plot through the *rms* package were used for calibration capability analysis and decision curve analysis (DCA) through the *nricens* package for determining the net clinical benefits. Differences were considered statistically significant at *P* < 0.05.

## Results

### Baseline characteristics of the study participants

Overall, 229 patients were enrolled in this study. Table 1 lists the baseline characteristics of subjects with and without MAFLD. Individuals with MAFLD had higher levels of BMI, ALT, AST, FPG, UREA, CREA, Cys-C, URIC, TG, TC, ALP, GGT, LDH, HBDH, non-HDL-C, TyG, TyG-BMI, and lower AST/ALT, TP, ALB, GLB, and HDL-C levels (all *P* < 0.05). As a result, the ratios of the above indicators, such as LDL-C/HDL-C, non-HDL-C/HDL-C, TG/HDL-C, and TC/HDL-C were greater in MAFLD patients than in the non-MAFLD group.

**Table 1:** The basic characteristics of the Non-MAFLD and MAFLD groups

	Non-MAFLD (*n* = 132)	MAFLD (*n* = 97)	*P* value
Age (years)	38.93 ± 9.51	42.59 ± 12.64	0.019*
Male (%)	60.6	77.3	0.008**
BMI (kg/m^2^)	22.87 ± 2.92	27.68 ± 3.53	< 0.001*
TBIL (μmol/L)	12.97 ± 4.73	13.77 ± 6.99	0.918*
DBIL (μmol/L)	3.74 ± 1.37	3.83 ± 1.95	0.597*
IBIL (μmol/L)	9.11 ± 3.50	10.45 ± 7.09	0.606*
ALT (IU/L)	20.11 ± 9.69	60.25 ± 44.58	< 0.001*
AST (IU/L)	20.04 ± 5.38	38.30 ± 24.70	< 0.001*
AST/ALT	1.15 ± 0.49	0.74 ± 0.29	< 0.001*
TP (g/L)	76.10 ± 3.35	74.74 ± 4.48	**0.010**
ALB (g/L)	48.91 ± 2.40	48.17 ± 2.78	**0.033**
GLB (g/L)	27.19 ± 2.87	27.05 ± 7.00	0.012*
ALB/GLB	1.82 ± 0.24	1.85 ± 0.35	0.111*
FPG (mmol/L)	4.89 ± 0.36	6.56 ± 2.78	< 0.001*
UREA (mmol/L)	4.57 ± 1.03	5.06 ± 1.64	0.015*
CREA (mmol/L)	69.78 ± 14.50	78.37 ± 20.56	0.001*
Cys-C (mg/L)	0.80 ± 0.08	0.88 ± 0.19	< 0.001*
URIC (μmol/L)	329.10 ± 65.94	393.14 ± 102.24	**< 0.001**
TG (mmol/L)	1.10 ± 0.48	2.81 ± 1.78	< 0.001*
TC (mmol/L)	4.59 ± 0.66	4.87 ± 1.02	**0.011**
HDL-C (mmol/L)	1.51 ± 0.41	1.03 ± 0.24	< 0.001*
LDL-C (mmol/L)	2.77 ± 0.61	2.87 ± 0.84	0.284
ALP (IU/L)	72.25 ± 19.23	85.69 ± 32.00	< 0.001*
GGT (IU/L)	23.61 ± 17.46	69.19 ± 64.38	< 0.001*
CK (IU/L)	133.11 ± 179.06	116.90 ± 60.95	0.586*
LDH (IU/L)	173.40 ± 26.34	194.08 ± 39.18	< 0.001*
HBDH (IU/L)	133.32 ± 19.58	143.60 ± 28.57	0.003*
nonHDL-C (mmol/L)	3.07 ± 0.71	3.84 ± 1.00	**< 0.001**
LDL-C/HDL-C	2.03 ± 1.07	2.86 ± 0.84	< 0.001*
nonHDL-C/HDL-C	2.29 ± 1.42	3.91 ± 1.29	< 0.001*
TG/HDL-C	0.85 ± 0.70	3.04 ± 2.43	< 0.001*
TC/HDL-C	3.29 ± 1.42	4.91 ± 1.29	< 0.001*
TyG	8.29 ± 0.40	9.40 ± 0.65	< 0.001*
TyG-BMI	189.87 ± 28.31	260.07 ± 38.91	< 0.001*

Data were expressed as mean ± SD. *P* value < 0.05 were considered statistically significant and indicated in bold

*using Mann-Whitney U test, **using Pearson’s chi-square test, others using Student’s t-test

### Variable selection and model construction

Among these indexes, 28 variables showed significant differences between the MAFLD and non-MAFLD groups. The differences in 17 variables remained after adjusting for a variety of factors, including age, sex, and BMI (Table 2).

**Table 2:** Logistic regression analysis of risk factors for MAFLD patients after adjusting age, gender, and BMI

Index	OR	95% CI	*P* Value
ALT	1.121	(1.075, 1.169)	< 0.001
AST	1.206	(1.124, 1.293)	< 0.001
A/A	.023	(0.005, 0.098)	< 0.001
FPG	8.408	(3.655, 19.341)	< 0.001
Cys-C	122.475	(3.920, 3826.590)	0.006
URIC	1.009	(1.004, 1.014)	< 0.001
TG	15.827	(6.209, 40.344)	< 0.001
HDLC	.009	(0.002, 0.047)	< 0.001
ALP	1.022	(1.005, 1.040)	0.012
GGT	1.055	(1.032, 1.078)	< 0.001
nonHDL-C	2.228	(1.429, 3.475)	< 0.001
LDL-C/HDL-C	1.796	(1.175, 2.745)	0.007
nonHDL-C/HDL-C	2.188	(1.555, 3.078)	< 0.001
TG/HDL-C	5.629	(3.039, 10.424)	< 0.001
TC/HDL-C	2.188	(1.555, 3.078)	< 0.001
TyG	182.474	(33.518, 993.407)	< 0.001
TyG-BMI	1.215	(1.142, 1.293)	< 0.001

**Fig. 1:**
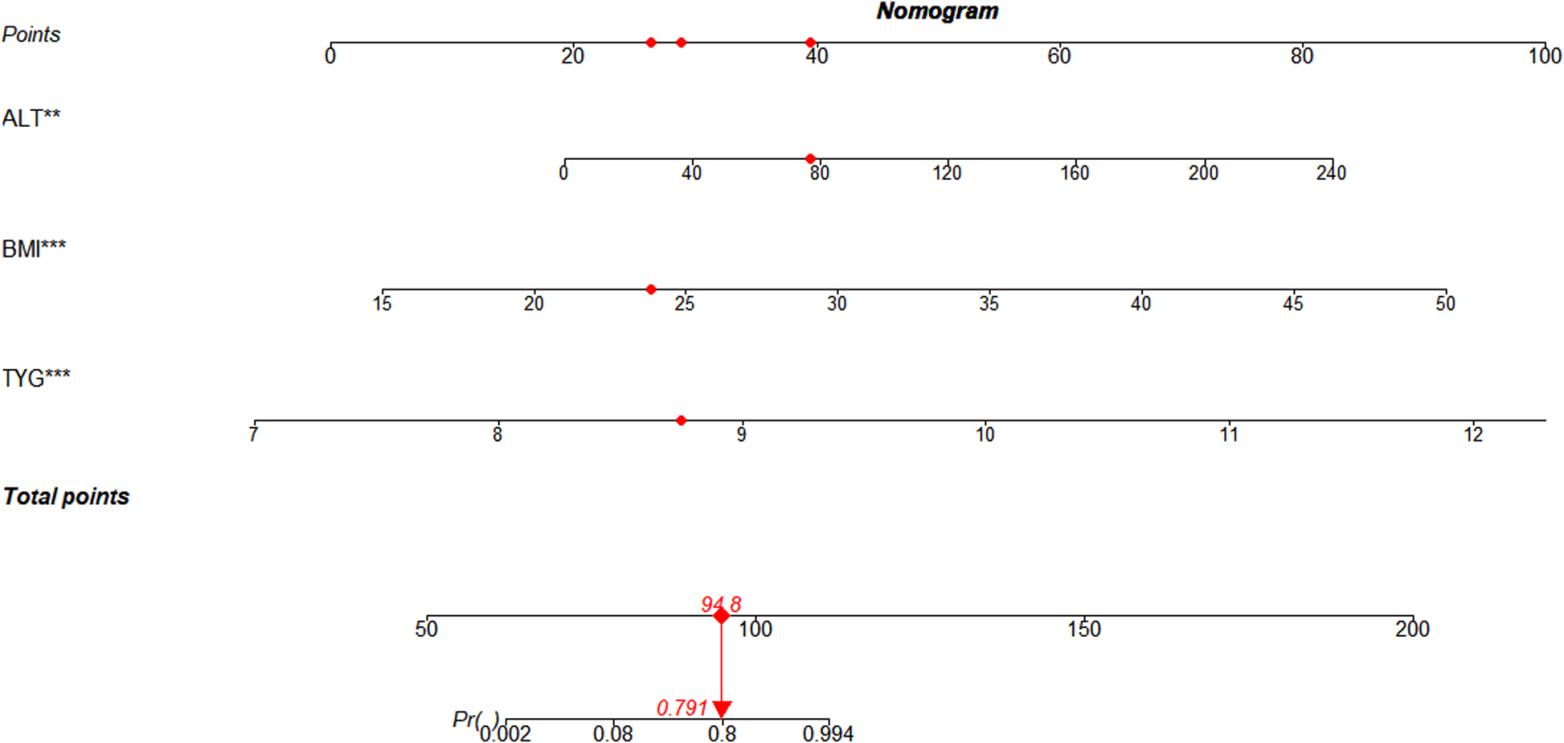
Dynamic nomogram of the diagnostic model

After all the above 17 variables, age, gender and BMI were included in binary logistic regression, the remaining 5 variables showed statistical differences, namely BMI, ALT, TG, TyG, and TyG-BMI. In order to strictly control the collinearity of variables, Variance Inflation Factor (VIF) was controlled below 2.5 [29]. Finally, a predictive model consisting of BMI, ALT, and TyG was constructed. The logistic regression model (Model) is expressed as 1/(1+ e^-(− 57.472 + 0.576*BMI + 0.061*ALT+ 4.15*TyG)) and the result of the Model means the probability of a diagnosis of MAFLD ranging from 0 to 1. As shown in Fig. 1, a dynamic nomogram was used to describe the probability of the MAFLD in the Model. For example, a patient with a BMI of 23.9 kg/m^2^, ALT of 77 IU/L, and TyG of 8.75 had a likelihood of MAFLD of 0.791 based on the Model.

### Diagnostic performance of vital indexes and the Model in MAFLD

**Fig. 2:**
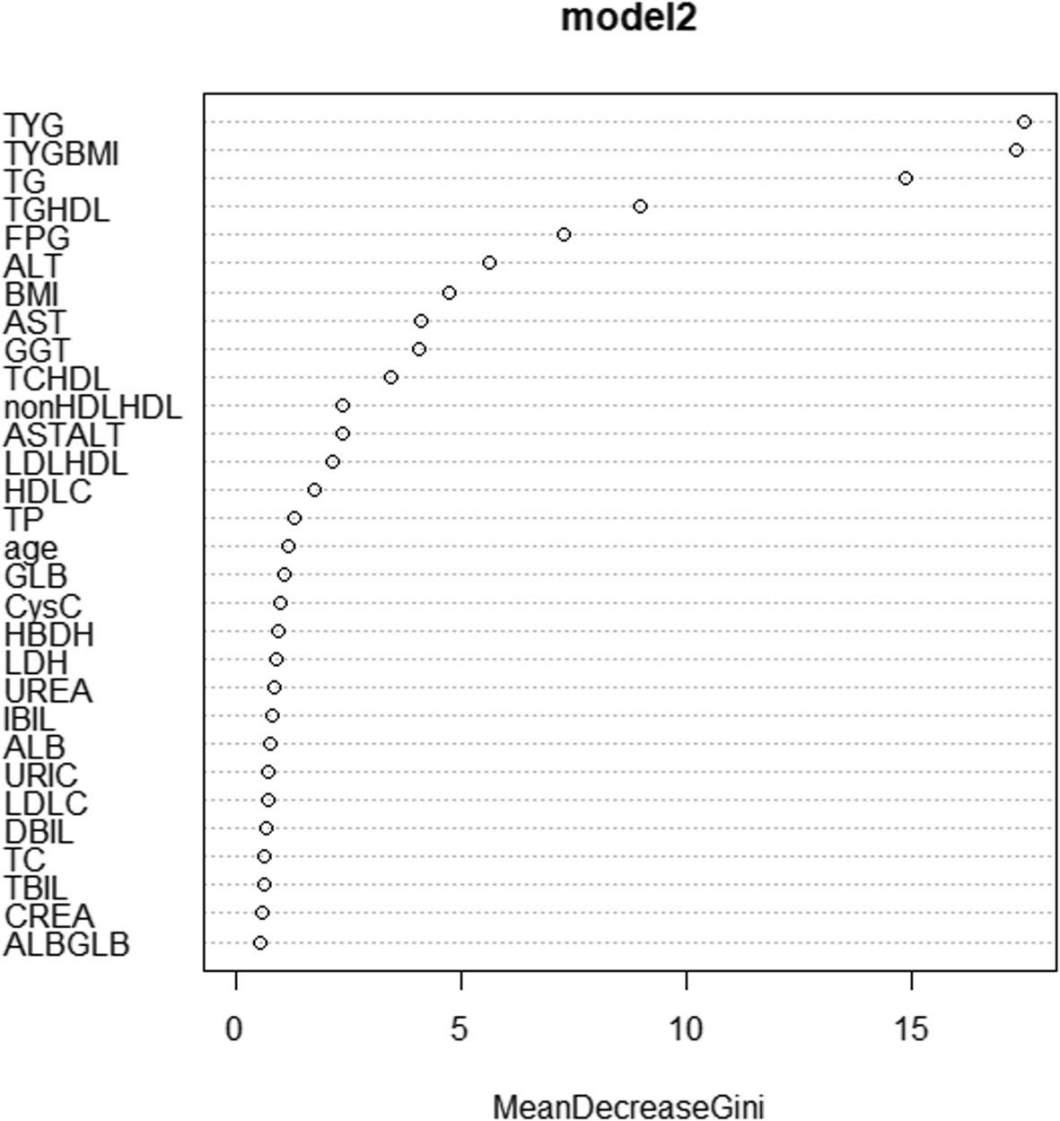
Importance score of different variable

When all the variables were adopted, a graph provided an overview of the importance score of each variable in predicting MAFLD by using the random forest method. The Mean Decrease in Gini is a measure to estimate the importance of the target variable. The higher the value is, the higher the importance of the variable is. As shown in Fig. 2, TyG, TyG-BMI, TG, and TG/HDL-C are the four most important indicators for identifying MAFLD.

**Fig. 3:**
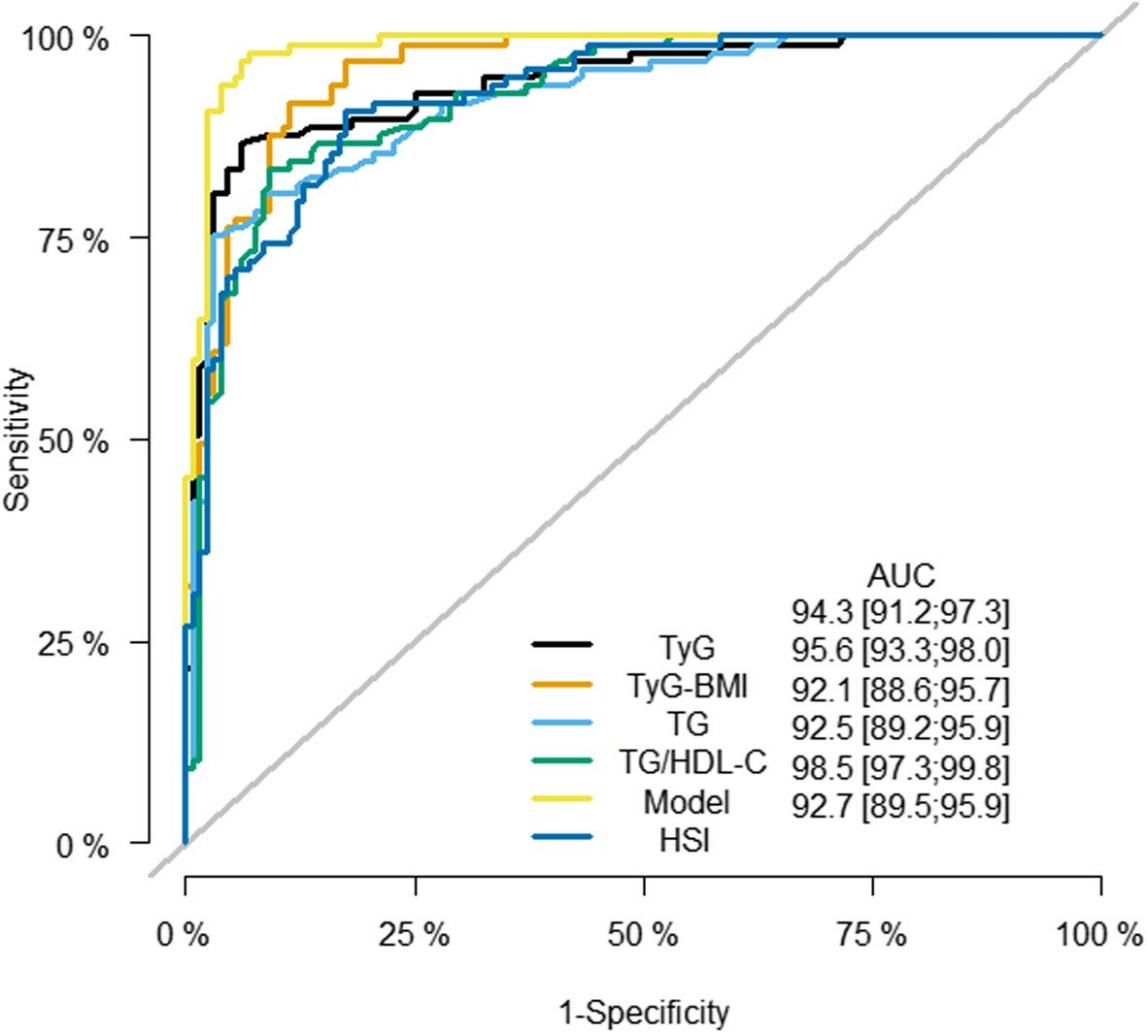
ROC curves of TG, TG/HDL-C, TyG, TyG-BMI, HSI, and the Model for MAFLD

To compare the performance of the four indexes and the new Model with HSI in diagnosing MAFLD, ROC curve analyses were conducted to identify the diagnostic value of TG, TG/HDL-C, TyG, TyG-BMI, HSI, and the Model (Fig. 3). As a result, the area under the receiver operating characteristic curve (AUROC) of the Model was 0.985 (95% CI 0.973–0.998) with 0.979 sensitivity and 0.932 specificity when the cut-off value was 0.985, showing the best capacity for assessing MAFLD. TG, TG/HDL-C, TyG, TyG-BMI, and HSI could also detect MAFLD as shown in Table 3.

**Table 3:** AUROC of TG, TG/HDL-C, TyG, TyG-BMI, HSI, and the Model for MAFLD

Variable	AUROC	95%CI	Cut-off value	Sensitivity (%)	Specificity (%)
TG	0.921	0.886 to 0.957	1.745	75.3	97.0
TG/HDL-C	0.925	0.892 to 0.959	1.260	83.5	90.9
TyG	0.943	0.912 to 0.973	8.805	86.6	93.9
TyG-BMI	0.956	0.933 to 0.980	221.585	91.8	88.6
HSI	0.927	0.895 to 0.959	35.275	90.7	82.6
Model	0.985	0.973 to 0.998	0.293	97.9	93.2

This study also compared the diagnostic efficacy of sex-specific cut-off point values of different indicators in the diagnosis of MAFLD. It could be seen from Table 4 that the cut-off values of TG, TG/HDL-C, TyG, TyG-BMI, HSI, and the Model in males were larger than that in females. As for males, the Model was the most sensitive to MAFLD diagnosis when the cut-off point value was 0.293, with a sensitivity of 100%. TG with a cut-off point value of 1.765 and TyG with a cut-off point value of 8.905 both had the same high specificity of 95% for MAFLD diagnosis. As for females, the sensitivity of the diagnosis of MAFLD was 100% when the cut-off values of TG/HDL-C and TyG-BMI were 0.765 and 207.96, respectively. The specificity in diagnosing MAFLD was 98.1% with a cut-off point value of 8.770 of TyG and 0.138 of the Model.

**Table 4:** AUROC of the sex-specific cut-point of TG, TG/HDL-C, TyG, TyG-BMI, HSI, and the Model for MAFLD

Variable	AUROC	95%CI	Cut-off value	Sensitivity (%)	Specificity (%)
Male					
TG	0.903	0.854 to 0.952	1.765	78.7	95.0
TG/HDL-C	0.906	0.857 to 0.954	1.300	86.7	85.0
TyG	0.926	0.882 to 0.969	8.905	85.3	95.0
TyG-BMI	0.945	0.912 to 0.978	226.885	90.7	87.5
HSI	0.913	0.870 to 0.957	36.950	77.3	90.0
Model	0.982	0.964 to 1.000	0.293	100.0	90.0
Female					
TG	0.953	0.904 to 1.000	1.250	95.4	86.5
TG/HDL-C	0.969	0.935 to 1.000	0.765	100.0	78.8
TyG	0.979	0.949 to 1.000	8.770	90.9	98.1
TyG-BMI	0.978	0.951 to 1.000	207.960	100.0	90.4
HSI	0.951	0.904 to 0.998	34.855	95.5	90.4
Model	0.996	0.987 to 1.000	0.138	95.5	98.1

As for the Model, five-fold cross-validation was used to describe its predictive efficacy for MAFLD. The values of RMSE (Root Mean Squared Error), MAE (mean absolute error), and R-squared were 0.3008045, 0.2404456, and 0.6548626 respectively. R-squared, also called the coefficient of determination, was used to indicate how well a model fits. The closer the R-squared value was to 1, the better the model discrimination was, and the closer to 0, the worse the model discrimination was. The value of this Model was 0.6548626, indicating that the Model fitted well.

**Fig. 4:**
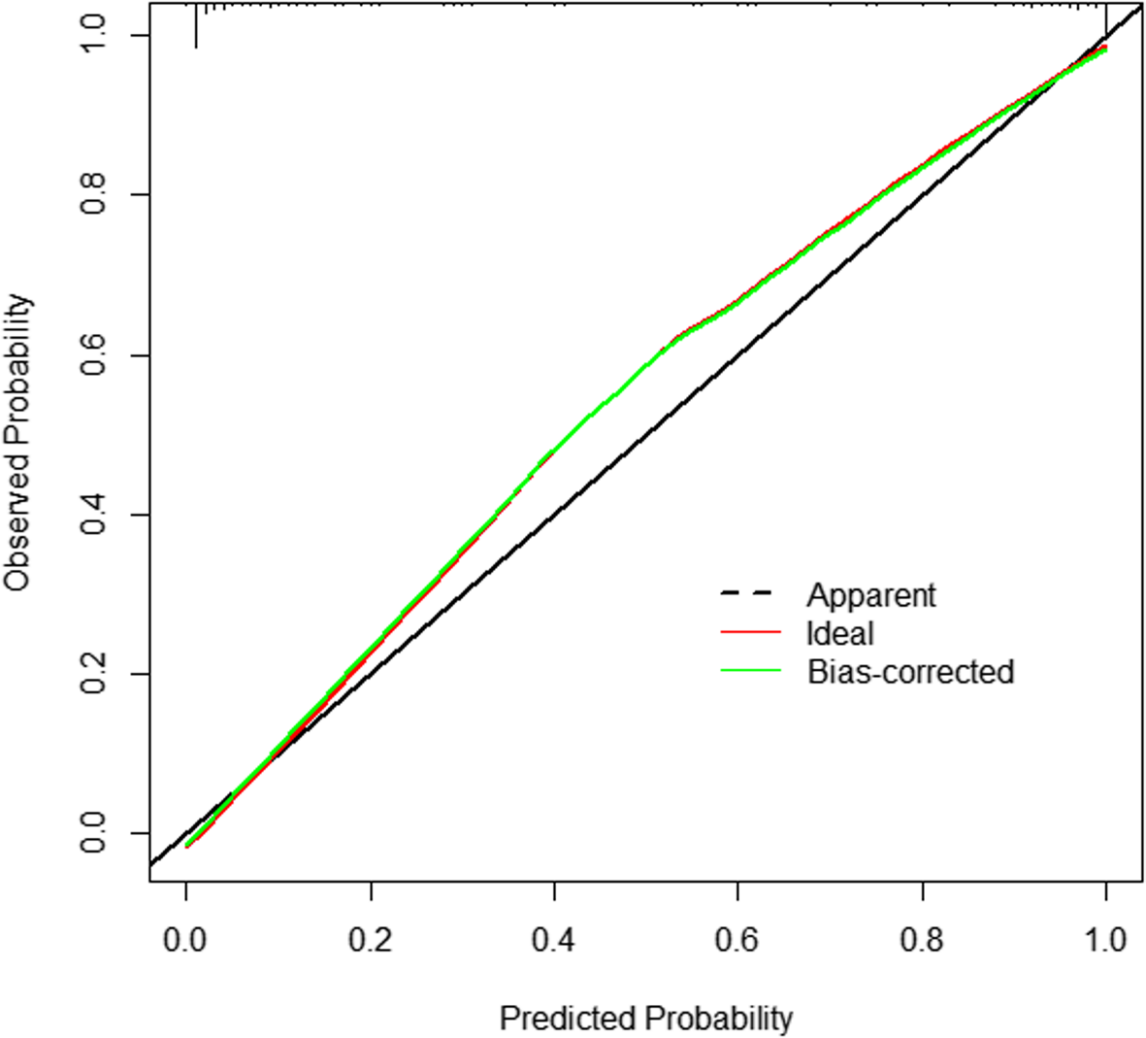
Calibration plot of the model

**Fig. 5:**
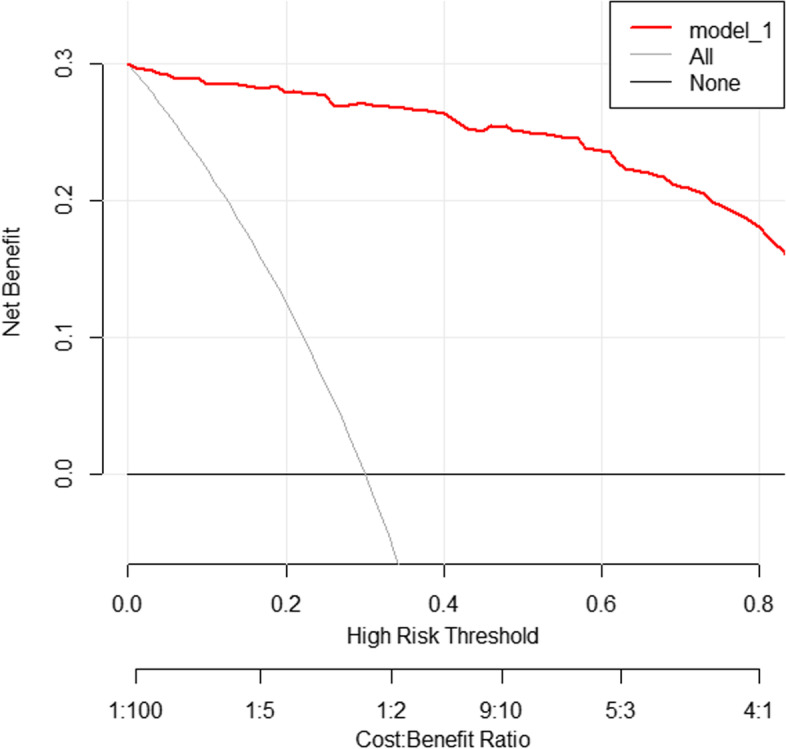
DCA curves of the model

As for calibration capability, the *P*-value of the Hosmer-Lemeshow test of the predictive model was 0.926, indicating that the difference between the predicted value and the observed value was not statistically significant, and the Model had good calibration ability. The calibration plot and DCA curves of the model are shown in Figs. 4 and 5, respectively. In the calibration plot, the abscissa is the probability predicted by the model, and the ordinate is the probability of the real event. The closer the calibration curve (called bias-corrected) was to the ideal curve (called ideal), the better the prediction ability of the Model was. As a result, the Model had good calibration ability.

The utility of the Model was verified by quantifying the net benefit under different risk thresholds. “None” indicated that all patients were non-MAFLD, “All” indicated that all patients were MAFLD, and “model_1” indicated the Model for diagnosing MAFLD. The red line was higher than the other two curves, which meant that the clinical benefit could be improved by using the Model to diagnose MAFLD.

## Discussion

This retrospective cross-sectional study assessed the ability of TG, TG/HDL-C, TyG, and TyG-BMI to predict MAFLD and constructed a novel model for diagnosing MAFLD. TyG-BMI performed the best among the four indices. The sensitivity and specificity of the Model based on BMI, ALT, and TyG improved to more than 90%. The most sensitive indicators for the diagnosis of MAFLD were the Model in males and TG/HDL-C and TyG-BMI in females. The most specific indicators for the diagnosis of MAFLD were TG and TyG in males and TyG and the Model in women. It was apparent from the Hosmer-Lemeshow test, five-fold cross-validation, and the pictures of the calibration plot and DCA curve that the Model fitted well and could improve the net benefit.

About 7.4% of MAFLD patients develop liver fibrosis [2]. In addition, MAFLD is also an increasing cause of hepatocellular carcinoma (HCC). With MAFLD-HCC, patients were older and had shorter survival times and more advanced tumors [30]. MAFLD patients were found to have an increased risk of hypertension [31]. Therefore, the identification of MAFLD is of vital importance. Liver biopsy is still the gold standard for MAFLD diagnosis, although it has a variety of associated risks, such as bleeding, pain, and death [32, 33]. In addition, sampling errors and sampling biases exist [34]. Noninvasive biomarkers that are cheap and effective for diagnosing MAFLD have been studied.

In MAFLD, TG accumulates in the liver and blood. Interestingly, TG/HDL-C and TyG, which are surrogate IR markers, are both derived from triglycerides. Independent of age, BMI, and waist circumference, it was observed that the expression of TG/HDL-C was higher in fatty liver patients with normal or even higher levels of ALT [35]. A cross-sectional study involving 18,061 physical examination patients from China found that TG/HDL-C can be considered as a risk factor and even a predictor of MAFLD with a lower cut-off value (0.9 vs. 1.4) and a greater AUROC (0.85 [0.84–0.86] vs. 0.79 [0.78–0.80]) in women than in men [36]. Similar to the previous study, this study found that the cut-off value of TG/HDL-C in diagnosing MAFLD was lower in women than in men (0.765 vs. 1.300) and higher in AUROC (0.969 [0.935–1.000] vs. 0.906 [0.857–0.954]). Besides, a follow-up study of non-obese people in China with normal blood lipids exhibited that the cut-off values of TG/HDL-C in diagnosing MAFLD were 0.69 and 0.65 for women and men [37]. In Japan, a cohort study of patients with more than 10 years of follow-up duration discovered the optimal cut-off points of TG/HDL-C for MAFLD diagnosis were 0.64 and 0.88 in women and men, respectively [38]. In addition, in a randomized controlled study, a decrease in TG and TG/HDL-C was associated with the resolution of non-alcoholic steatohepatitis (NASH) [39]. In another study, TC/HDL-C, a lipid ratio parameter, was used to diagnose MAFLD with an AUROC of 0.645 [40]. Both higher levels of TC/HDL-C and TG/HDL-C indicated more severe liver steatosis, while the former showed higher specificity [41]. It was previously shown that TC/HDL-C, TG/HDL-C, LDL-C/HDL-C, and non-HDL-C/HDL-C were positively related to the severity of hepatic steatosis [42]. From the results of this research, all of the above indexes were risk factors for MAFLD independent of age, sex, and BMI.

A high level of TyG means a patient has high levels of TG or FPG. However, in IR patients, glucose uptake in fat and muscle tissue is decreased and fat accumulation in the liver is increased. As a result, the increase of TG and FPG leads to an increase of TyG [43]. Compared with HIEC and HOMA-IR, TyG can be used as an effective surrogate indicator of IR [19, 44, 45]. TyG index was also found to be related to damage of large vessels and microvessels [46]. Some studies had found that TyG was a risk factor for metabolic diseases such as hypertension, diabetes, and coronary heart disease, and could be used to predict the risk of CVD [47–50]. Previous studies have shown that the incidence of MAFLD also significantly increases with the increase in TyG [51, 52]. In other studies, the AUCs of the TyG index to detect adult MAFLD were 0.760–0.782, with MAFLD diagnosed by ultrasound [53–56]. A study showed that the AUC of TyG was larger than that of ALT, with the AUC of TyG at 0.9 (95%CI 0.84–0.94) in biopsy-proven MAFLD with 80% sensitivity and 92% specificity when the cut-off value was 8.38 [57]. Similarly, the AUC of TyG in diagnosing MAFLD in this study was 0.943 (95%CI 0.912–0.973) with 86.6% sensitivity and 93.9% specificity when the cut-off value was 8.805. Furthermore, among 50 asymptomatic women who underwent a liver biopsy, TyG displayed high sensitivity in screening simple steatosis and NASH [58]. Based on a large number of MAFLD participants (*n* = 11,424) in a follow-up study, it was found that patients with a high TyG index were more likely to have MAFLD progression [59]. TyG-BMI, a modified TyG index, showed a better ability to identify MAFLD than TyG itself, not only in males but also in females. The AUROC of TyG-BMI in diagnosing MAFLD ranged from 0.774–0.9084 [60–62]. In this study, it was discovered that the AUROC of TyG-BMI was 0.956 (95% CI 0.933–0.980), which was higher than that reported in other studies. As for non-obese subjects, TyG-BMI could identify MAFLD and predict its occurrence effectively [63, 64]. In addition to liver steatosis, TyG and its related marker TyG-BMI were found to be related to liver fibrosis [56, 65].

HSI was made up of indexes chosen by multivariate logistic analysis of the derived cohort (2680 NAFLD and 2680 non-NAFLD patients). The AUROC of HSI for diagnosis of NAFLD was 0.812 (95%CI 0.801–0.824). In the validation cohort (2682 NAFLD and 2682 non-NAFLD patients), the AUROC of NAFLD was 0.819 (95%CI 0.808–0.830) [66]. It was found that the AUROC of HSI (0.8678 (95%CI 0.8604–0.8752)) in diagnosing NAFLD was higher than TyG (0.8084 (95%CI 0.7996–0.8173)) and TG/HDL-C (0.8147 (95%CI 0.8060–0.8233)), lower than TyG-BMI (0.8862 (95%CI 0.8777–0.8927)) [61]. In this study, the AUROC of HSI (0.927 (95%CI 0.895–0.959)) in diagnosing MAFLD was higher than TG/HDL-C (0.925 (95%CI 0.892–0.959)), and lower than TyG (0.943 (95%CI 0.912–0.973)), TyG-BMI (0.956 (95%CI 0.933–0.980)) and the Model (0.985 (95%CI 0.973–0.998)). The diagnostic value of indicators such as TG/HDL-C, TyG, and TyG-BMI in the study was higher than that of other studies mentioned above, which may be due to a small number of samples and the lack of suspected cases of MAFLD.

### Strength and limitations

Previous studies demonstrated that TyG and TG/HDL-C were increased in MAFLD patients and could be used as diagnostic markers. The current study compared these two IR-related indicators and found that both had good diagnostic efficacy for MAFLD. Based on this, a better diagnostic model was established. This study has several limitations. First, major limitations of the study include a small sample size and the use of liver ultrasound as a gold-standard to evaluate the accuracy of the two scores. Ultrasound detection of steatosis less than 20% has limited sensitivity. It also performs poorly when differentiating between steatosis and fibrosis and is inconsistent. Furthermore, the detection capability is highly dependent on the examiner [67]. Second, the relationships between TG/HDL-C, TyG, and HOMA-IR aren’t verified in this research. Third, to measure the diagnostic significance of the indicators for MAFLD more accurately, the suspected population of MAFLD should be included; otherwise, the value of these indicators will be exaggerated. Finally, the basic of machine learning is the task of dividing the dataset into training and testing sets to validate the model. However, due to the difficulty of the cumulation of data in the medical field, this work was replaced into hypothesis work, such as *p*-value or confidence interval.

## Conclusion

This study compared the ability of two surrogate indices for IR, TG/HDL-C, and TyG, in predicting MAFLD for the first time. TyG and TG/HDL-C can be easily measured with high accuracy and is low cost because TG, FPG, and HDL-C can be conveniently measured. When the cut-off point value was 8.805, the sensitivity and specificity of TyG in the diagnosis of MAFLD were higher than TG/HDL-C. In addition, the combination of BMI, ALT, and TyG could improve the diagnosis of MAFLD. This study suggested that overweight person with abnormal glycolipid metabolism and liver function were more likely to develop MAFLD, which is conducive to promoting the screening and management of MAFLD.

## Data Availability

The datasets used and analyzed during the current study are available from the corresponding author on reasonable request.

## Abbreviations

TyG: Triglyceride and glucose index
TG/HDL-C: Triglyceride-to-high density lipoprotein cholesterol ratio
IR: Insulin resistance
MAFLD: Metabolic-Associated Fatty Liver Disease
ALT: Alanine aminotransferase
AST: Aspartate aminotransferase
A/A: AST/ALT
GGT: Gamma-glutamyl transferase
ALP: Alkaline phosphatase
FPG: Fasting plasma glucose
TC: Total cholesterol
TG: Triglyceride
HDL-C: High-density lipoprotein cholesterol
LDL-C: Low-density lipoprotein cholesterol
URIC: Uric acid
Cys-C: Cystatin C
NAFLD: Nonalcoholic Fatty Liver Disease
NASH: Nonalcoholic steatohepatitis
T2DM: Type 2 diabetes mellitus
AUROC: Area under receivers operating characteristic curve
BMI: Body mass index
HOMA-IR: Homeostatic model assessment of insulin resistance
